# An efficient Terahertz rectifier on the graphene/SiC materials platform

**DOI:** 10.1038/s41598-019-47606-6

**Published:** 2019-08-01

**Authors:** Maria T. Schlecht, Sascha Preu, Stefan Malzer, Heiko B. Weber

**Affiliations:** 1 Friedrich-Alexander University of Erlangen-Nürnberg (FAU), Applied Physics, Staudtstr. 7/A3, 91058 Erlangen, Germany; 2 Department of Electrical Engineering and Information Technology, Technical University Darmstadt, Merckstrasse 25, 64283 Darmstadt, Germany

**Keywords:** Electronic properties and devices, Terahertz optics, Electronic devices

## Abstract

We present an efficient Schottky-diode detection scheme for Terahertz (THz) radiation, implemented on the material system epitaxial graphene on silicon carbide (SiC). It employs SiC as semiconductor and graphene as metal, with an epitaxially defined interface. For first prototypes, we report on broadband operation up to 580 GHz, limited only by the RC circuitry, with a responsivity of 1.1 A/W. Remarkably, the voltage dependence of the THz responsivity displays no deviations from DC responsivity, which encourages using this transparent device for exploring the high frequency limits of Schottky rectification in the optical regime. The performance of the detector is demonstrated by resolving sharp spectroscopic features of ethanol and acetone in a THz transmission experiment.

## Introduction

Silicon carbide (SiC), a wide-bandgap semiconductor with outstanding properties, evolves into a major platform for novel physics and technology. Beyond its leading role in high-power electronics and as substrate for GaN devices^1^, the use of epitaxial graphene on its (0001) facet^2^ allows for a wealth of experiments including functional electronics^3–7^, single-molecule electronics^8^, plasmonics and phononics^9–12^, and detection of ultra-fast electronic processes^13^. Further, powerful novel quantum systems as single-photon sources^14–17^, spin systems^18,19^, and high-finesse optomechanics^20,21^ can be established. Additionally, a variety of biological and chemical sensors can be manufactured from the material system SiC/graphene^22–24^. Key to many applications is the strict sp^3^ bonding scheme of the SiC that provides robustness similar to diamond and also its excellent heat conduction^25^. In particular, we have recently demonstrated ultra-fast photocurrents in SiC devices from which we expect THz generation within a SiC wafer^26^. SiC is transparent in the THz regime up to 11.3 THz^27,28^ and has a high refractive index. In order to avoid further lossy interfaces it is desirable to provide a THz detection scheme on the very same chip that is comparable in sensitivity to state-of-the-art detectors^29–31^.

Schottky diodes are well suited for the detection of THz radiation due to their high sensitivity, their reliability, their large bandwidth and their short response times^32–34^. They find applications in the field of THz imaging and THz spectroscopy which proved to be useful methods for the detection of drugs^35^ and explosives^36^. Furthermore, THz systems are used for industrial quality control^37,38^. Here, the THz range is advantageous compared to X-rays as it is non-destructive and non-ionizing.

In this paper we present a THz detecting device on the material platform graphene/SiC which is based on a metal-semiconductor Schottky diode. It comprises an epitaxially grown graphene layer^2^ as the metal on the (0001) facet of 4H SiC, which serves as n-type semiconductor as well as semi-insulating substrate. The energy alignment is controlled with epitaxial precision^39,40^.

## Device Concept

**Figure 1:**
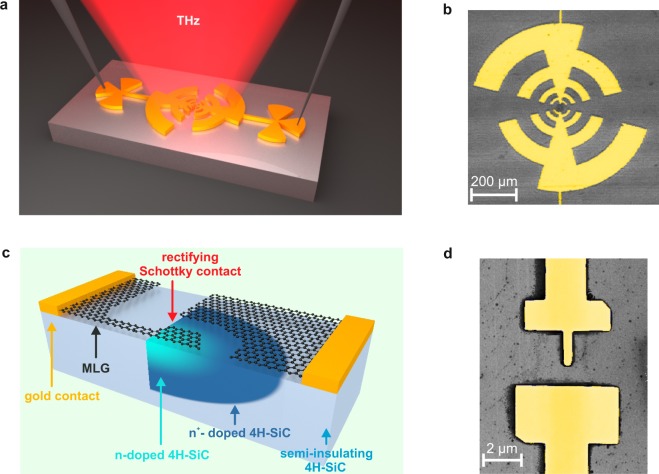
Schematic of the device and the corresponding SEM images. (**a**) The Schottky diode is connected to a logarithmic periodic antenna. The THz radiation is transformed by the antenna into a THz voltage which is rectified by the device. The resulting time-averaged current *I*_*DC*_ is proportional to the square of the field amplitude of the THz radiation. (**b**) Corresponding colored SEM image of a logarithmic periodic antenna. (**c**) Close-up of the Schottky diode. MLG forms the metal of the Schottky contact, n-doped SiC the semiconductor. By increasing the doping level, an ohmic contact between the two materials is achieved. (**d**) Corresponding colored SEM image of the device.

As compared to previous MESFET designs which used intercalated graphene gates, optimized for high on/off ratios^6^, we used a simplified device concept which employs the as grown interface between epitaxial graphene and 4H-SiC providing a Schottky barrier height of 0.35 eV. The result is a fully transparent Schottky diode with near-zero-bias operation capability. For our THz detection scheme a Schottky diode with an area of 0.4 *μ*m^2^ is centered between two metallic antenna arms such that THz radiation from free space is coupled in and is transformed to an oscillating voltage at the diode (cf. Fig. 1) which in turn rectifies the AC bias and delivers a DC current proportional to the received THz power^41^. In this layout, one metallic antenna arm is in close contact with the graphene surface and the underlying Schottky contact. There is a 1 *μ*m gap to the opposite antenna arm that is coupled via an ohmic contact to the active semiconductor area (Fig. 1d). This latter contact is formed by the very same combination of graphene/SiC, but is at a significantly higher doping level, achieved by contact implantation^39^.

**Figure 2:**
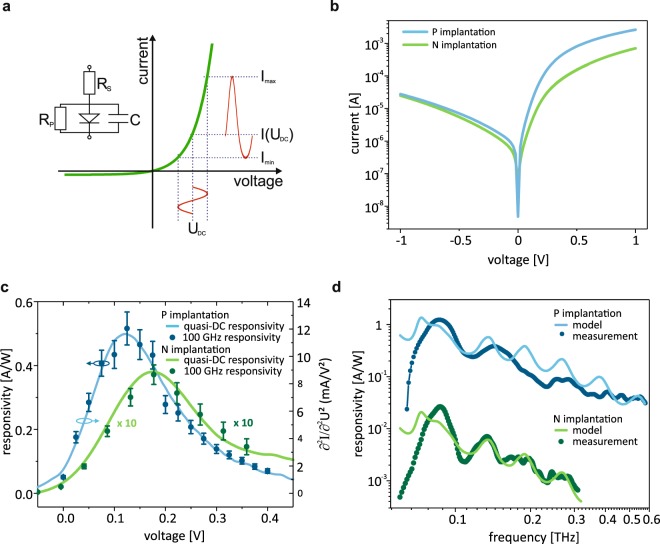
DC characteristics and THz responsivity. (**a**) Schematic of rectification in a Schottky diode and its equivalent circuit. An incident *U*_*THz*_ at a bias voltage *U*_*DC*_ translates via the curvature into an asymmetric AC current response that increases the time-averaged (DC) current. (**b**) DC IV-characteristics of two diodes. (**c**) DC responsivities (full lines) and THz responsivities (at 100 GHz, symbols) as a function of bias voltage. (**d**) Responsivities as a function of incident THz frequencies at the responsivity peak of (**c**). A Fourier filter was applied to suppress Fabry-Perot interference due to resonant pathways of the setup. Solid lines represent electrodynamical calculations that includes the ideal antenna characteristics and the RC roll off of the circuit, showing excellent agreement. The phosphorous device exposes deviations due to known antenna imperfections of this particular device. A second test device with little deviations is shown in the Supplementary Information.

The rectification of the AC voltage excitation into a net DC current generates the detector signal^42^. The detection principle is displayed in Fig. 2a: the THz voltage ${U}_{THz}(t)={\hat{u}}_{THz}\,\cos ({\omega }_{THz}t)$ adds to the DC bias *U*_*DC*_. As a consequence of the non-linearity of the IV-characteristics it results in a time averaged DC current ${\langle {I}_{THz}\rangle }_{DC}=\frac{1}{2}\frac{{\partial }^{2}I}{\partial {U}^{2}}{\hat{u}}_{THz}^{2}$ in the small signal limit. The figure of merit for the THz detector is the responsivity and the noise floor. The responsivity is defined as the ratio of the detected DC current level and the THz power incident to the antenna. It is therefore a function of applied DC voltage and the received  THz frequency. Further, we varied device parameters by using different doping concentrations, geometry, antenna design and finally, for comparison, by substituting graphene by nickel.

## THz Detection

First, the standard n-type dopant for SiC, nitrogen, is used, with a doping concentration of 1 · 10^19^ *cm*^−3^ beneath the ohmic contact. The resulting DC IV-characteristics, shown in Fig. 2b (green), has a typical diode shape $I(U)={I}_{0}(\exp (\frac{eU}{n{k}_{B}T})-1)$^43^ with a saturation current *I*_0_ ≈ 300 nA and an ideality factor *n* ≈ 1.7. The Schottky barrier height *ϕ*_*B*_ can be derived from these parameters according to ${\varphi }_{B}=\frac{{k}_{B}T}{n\,e}\,\mathrm{ln}(\frac{{A}^{\ast }{T}^{2}}{{I}_{0}})$ where A^*^ is the effective Richardson constant^44^. The numerical second derivative of the IV curve is displayed in Fig. 2c, showing a pronounced maximum that is a result of the interplay of exponential increase and subsequent current limitation by the serial resistance *R*_*S*_. When used as a detector, the responsivity at the maximum of Fig. 2c is shown as a function of frequency in Fig. 2d. Not surprisingly, we observe an RC roll off with a 3dB frequency of 35 GHz, indicating an unfavorably high serial resistance of *R*_*S*_ ≈ 0.7 kΩ, determined by a linear fit to the IV-characteristics at high forward bias. For the present device, this can be assigned to the high sheet resistance of the semiconductor. In order to circumvent this limitation, we opted for the rather unconventional phosphorous dopant, which gives access to 1 · 10^20^ *cm*^−3^ doping levels. Such devices show two significant improvements (see Fig. 2b,c): first, the DC IV-characteristics appears to be steeper in the forward direction indicating an ideality factor that is closer to 1, namely *n* ≈ 1.2. Second, the serial resistance was reduced to *R*_*S*_ ≈ 0.25 kΩ which shifts the 3dB RC frequency to about 100 GHz. Both parameters contribute favorably to a much more pronounced maximum of the second derivative $\frac{{\partial }^{2}I}{\partial {U}^{2}}$ (cf. Fig. 2c), and, consequently, to the responsivity. Altogether, the responsivity, displayed in Fig. 2d is significantly improved for phosphorus implanted devices, with a maximum of 1.1 A/W at 90 GHz, which corresponds to state of the art devices^29–31^. A second device with only slightly lower performance data is displayed in the Supplementary Information.

It is instructive to trace the responsivity as a function of the DC bias. Figure 2c shows a comparison of DC responsivity which reflects the statically determined IV-characteristics (full lines). The symbols, in contrast, are the experimentally determined responsivities at approximately 100 GHz. One can see immediately that they essentially coincide (with a proportionality factor of ≈40 Ω that originates from the radiation resistance, the device resistance and taking into account various RF loss factors within the electrical circuit. Note that this impedance is only very weakly voltage dependent). This corroborates that the model we applied is fully appropriate. It further indicates that the quasi-static DC responsivity and those on the ps/THz time scale are essentially identical^45^.

## Comparison to Nickel/N-Doped SiC Schottky Diodes

**Figure 3:**
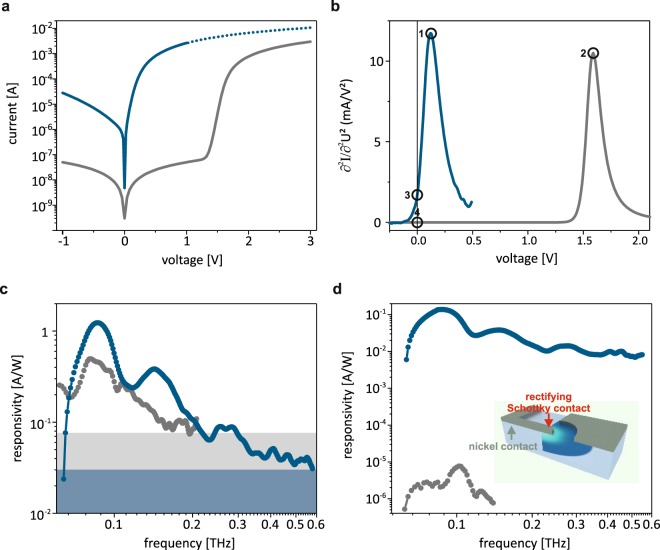
Comparison of graphene and nickel Schottky diodes. (**a**) DC IV-charateristics. The threshold voltage is significantly shifted to positive bias for the nickel diode. (**b**) Quasi-static DC-responsivities. Both device exhibit a similar peak value, but the graphene device provides the advantage of a higher zero-bias curvature. (**c**) Responsivity of the two devices at the peak positions 1 and 2 in (**b**) as a function of frequency. The shaded areas indicate the respective noise floor. (**d**) Responsivities of both devices at zero bias (position 3 and 4 in (**b**)). The graphene device remarkably outperforms the nickel device. Additionally, an artistic view of the nickel device is shown as an inset.

In order to elucidate the peculiarity of this device we performed comparisons with the state of the art material system for high performance Schottky devices within the SiC materials platform, nickel on n-type SiC. The fabrication effort is much more complex: In a first lithography step, nickel is deposited and subsequently annealed for the ohmic contact. In a second lithography step, nickel is deposited for the Schottky contact without annealing. All semiconductor parameters are kept identical to the graphene Schottky diode. The results are displayed in Fig. 3. First, we discuss the static IV-characteristics of the nickel Schottky device which displays excellently low dark current (see Fig. 3a), a fact that is, however, of little importance for the rectification. The key difference is that the graphene Schottky device has a much lower onset voltage compared to the nickel device which is a consequence of its lower Schottky barrier height (Ni: 1.7 eV, MLG: 0.35 eV, as determined from IV characteristics). The importance for the rectification functionality becomes apparent from Fig. 3b, in which the quasi-static DC-responsivity of the forward current is shown. The peaks are very similar, but shifted. On a first glance, the optimum operation point corresponds to the maximum of the second derivative that is 0.12 V for the graphene device and 1.7 V for the nickel device.

We now turn to the THz detection with both the nickel and the graphene devices. The responsivity at the peak positions **1** and **2** of the quasi-static DC-responsivities shows a higher value for the graphene device (by a factor of 2, see Fig. 3c). The noise floor can be seen as shaded areas in the same figure. Due to the appearance of the peak at much higher bias for the nickel device, its noise floor is substantially higher. For detection purposes zero bias operation is preferred, a simplification that reduces the noise floor and the complexity of the setup. Almost all commercial applications therefore use zero-bias operation^30,46–48^ where applicable. At this point of operation (**3** and **4**) the graphene device displays a value of 1.7 mA/V^2^ in the differential conductance, 10^4^ times larger than the nickel device. In the THz detection measurement, accordingly, we find responsivities which are four orders of magnitude higher than the nickel device (see Fig. 3d). We find a responsivity of 0.14 A/W at 90 GHz for the graphene-SiC device and 7.6 · 10^−6^ A/W at 100 GHz for the nickel device at zero bias.

## Gas Spectroscopy Measurements

**Figure 4:**
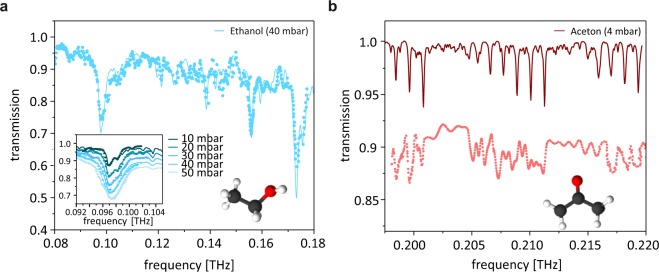
Transmission spectra of organic compounds in the gas phase. (**a**) Transmission spectrum of ethanol. Symbols represent measurements with the phosphorus implanted graphene Schottky diode, lines are calculated from the JPL database. The inset displays the pressure broadening of the 98 GHz feature which consists of a multiplet of lines. (**b**) Transmission spectrum of acetone in a narrow spectral range. The differences to calculations are not an experimental artifact, but indicate an insufficient database for the simulation.

To demonstrate the performance of our graphene/SiC THz detector we carried out spectroscopic absorption measurements of organic molecules. We opted for simple molecules, ethanol and acetone, which recently reattracted scientific attention when it was realized that they play a major role in everyday immission. It turned out that their ubiquitous occurance stemming from solvents exceeds the immission of fossil fuel exhausts, in particular indoors^49^. In our experiment the THz beam created by a nipnip photodiode^50^ was sent through a chamber filled with ethanol/acetone at low pressure **p** (1–150 mbar). In contrast to time domain THz spectroscopy this technique allows a point by point analysis of the transmission in frequency space with high spectral intensity (1 MHz spectral resolution, only limited by the linewidth of the implemented lasers). Figure 4a displays results obtained with ethanol in a spectral range from 80 GHz to 180 GHz, in which ethanol has rich spectral features. The displayed transmission spectra are normalized by a calibration measurement of an evacuated chamber. Our results (symbols) excellently reproduce the calculated spectra from the JPL database (line)^51^. The spectral fingerprints of the compound are nicely resolved. The inset displays a spectral feature at 98 GHz for various pressures. The calculated pressure broadening (lines) is accurately reproduced by our measurement (symbols). As a second example, acetone is investigated in the frequency range of 195 GHz to 220 GHz with high resolution. For this example we find substantial discrepancies between the calculations from the JPL database and our measurements: We find a broadband reduction of the overall intensity of about 10 percent for this particular pressure. Further, the spectral features occur at the calculated positions, but the overall shape of the spectrum has significant deviations. We stress that this is not an artifact of our detection setup as corroborated by the use of commercial detectors. Its origin rather lies in the preliminary and incomplete data of the JPL database. Note that the amplitude of the resolved lines are only a few percent, in some regions peaks with only 1 percent amplitude can be properly resolved. These measurements underscore that our detector is perfectly suited to resolve THz transmission spectra and recovers both agreement with calculated data in the case of ethanol, and meaningful differences in the case of acetone in the same setup.

Compared with a different, transistor-based detection scheme using exfoliated graphene on SiO_2_^52^, our detector offers significantly better performance and can be fabricated reliably on the mature material system SiC. We compare the noise equivalent power: 200 nW/$\sqrt{{\rm{Hz}}}$ for graphene:SiO_2_ transistors compared to 200 pW/$\sqrt{{\rm{Hz}}}$ at 90 GHz for phosphorus implanted Schottky diodes. Considering only thermal and current noise we would expect a NEP of approximately 5 pW/$\sqrt{{\rm{Hz}}}$. The origin of the noise is predominantly 1/f noise (evaluated at the chopping frequency). We were able to confirm that by measurements of the noise spectral density^53^ (Supplementary Information). Note that at this early stage there is plenty of room for improvements in particular for the capacitances and serial resistances. An impedance matched antenna would further increase the responsivity^33^.

## Conclusion

To conclude, we present a detection scheme for THz radiation on the SiC platform that benefits from the extraordinary properties of the epitaxial graphene/SiC interface. This metal-semiconductor contact allows for building a nearly transparent Schottky diode. Its low barrier is suited for zero bias detection, a quality that enables a simple circuitry and low-noise detection. We fitted this diode with a broadband metallic antenna, enabling THz detection up to 0.58 THz and a maximum responsivity of 1.1 A/W at 90 GHz. We performed spectroscopic measurements and resolved narrow, but weak spectral features of organic compounds. THz detection is a valuable device concept on the versatile SiC material platform that can be established side by side to single photon sources, high finesse optomechanics and graphene electronics. An astonishing observation is that electrical characteristics determined under DC conditions and THz measurements show accurately the same voltage dependence, indicating that the intrinsic high-frequency limitations are by far not reached. This, together with the almost complete transparency of these devices encourages further, unprecedented experiments: we anticipate that graphene/SiC Schottky diodes are well suited for exploring the intrinsic capabilities of Schottky rectification towards optical (Petahertz) frequencies.

## Methods

### Device fabrication

**Figure 5:**
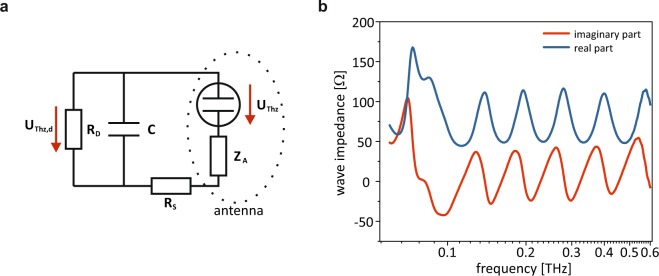
Theoretical model. (**a**) Equivalent circuit used to calculate the power lost due the impedance mismatch of the device and the antenna. (**b**) Radiation impedance of the logarithmic-periodic antenna.

A high purity, semi-insulating 4H-SiC wafer from Cree Inc. was partially n-doped by ion implantation. The implantation depth of the two dopants, nitrogen and phosphorus, was approximately 600 nm. At the designated Schottky contact the concentration was low at 1 · 10^17^ *cm*^−3^ (depth 200 nm). Below this Schottky contact we included a current spreading layer with a concentration of 1 · 10^19^ *cm*^−3^ in order to keep the sheet resistance of SiC low. For the ohmic contact the implantation dose was set to the same level as in the current spreading layer for N whereas the phosphorus concentration was increased to 1 · 10^20^ *cm*^−3^. The sample was heated to 600 °C during phosphorus implantation to reduce lattice damages. Subsequently, the samples were annealed at 1700 °C in argon atmosphere while the surface was protected by a carbon cap^54^. MLG was grown by thermal decomposition on the Si-terminated (0001) SiC-facet at 1625 °C under argon flux near normal pressure. The contact regions of the designated Schottky and ohmic contacts were defined by electron-beam lithography and evaporated with titanium/gold. The residual MLG was removed by oxygen plasma etching. After the IV characterization, the samples were attached to a logarithmic periodic broadband antenna, with an operation range from 60 GHz to 1500 GHz. The antenna characteristics simulated with CST are shown in Fig. 5b.

### Characterization

The electrical characterization of the Schottky diodes was performed using a HP4141 source measurement unit. For an efficient coupling of THz radiation from free space onto the Schottky diode it was mounted on a hyperhemispherical silicon lens. As a continuous THz source a n-i-pn-i-p superlattice photomixer was used with *μ*W power level^50^. It was imaged to the detector by two parabolic mirrors and detected using the Lock-in technique for noise suppression. We used a calibrated Golay cell as a reference for the detector measurements.

### Model derived from the IV characteristics

The detected current 〈*I*_*THz*_〉_*DC*_ is in the small signal limit proportional to the second derivative of the IV curve, i.e. its curvature.

1 $${\langle {I}_{THz}\rangle }_{DC}=\frac{1}{2}\frac{{\partial }^{2}I}{\partial {U}^{2}}{|}_{{U}_{DC}}{\hat{u}}_{THz,d}^{2}$$

${\hat{u}}_{THz,d}$ is the amplitude of the AC voltage at the diode due to the THz field. The maximum THz power which can be coupled to the antenna is ${P}_{THz}=\frac{{\hat{u}}_{THz}^{2}}{2{R}_{A}}$ where ${\hat{u}}_{THz}$ differs from the AC voltage ${\hat{u}}_{THz,d}$ at the diode due to the impedance mismatch of the antenna and the device. Therefore, the responsivity of the Schottky diode yields

2 $$ {\mathcal R} [\frac{A}{W}]=\frac{{\langle {I}_{THz}\rangle }_{DC}}{{P}_{THz}}={R}_{A}\frac{{\partial }^{2}I}{\partial {U}^{2}}{|}_{{U}_{DC}}{\eta }_{imp}$$

*η*_*imp*_ can be calculated considering the equivalent circuit of the whole device and the radiation impedance of the logarithmic-periodic antenna which are both shown in Fig. 5. Further aspects that decrease the AC voltage at the Schottky Diode are optical losses *η*_*opt*_ like reflection losses at the interface of the silicon lens and air and the RC roll off *η*_*RC*_

3 $${\eta }_{RC}=\frac{1}{1+\frac{{\nu }_{THz}^{2}}{{\nu }_{3dB}^{2}}}$$

with the 3dB frequency *ν*_3*dB*_ = 1/2*π*(*R*_*S*_ + *R*_*A*_)*C* depending on the serial resistance *R*_*S*_, the antenna resistance *R*_*A*_ and the capacitance *C*. The latter one can be modelled by a classical plate capacitor taking the edge capacitance into account.

4 $$C={\varepsilon }_{0}{\varepsilon }_{r}\frac{{A}_{D}}{{d}_{s}}+3{\varepsilon }_{0}{\varepsilon }_{r}\frac{{A}_{D}}{D}$$

Here *d*_*S*_ is the depletion length, *A*_*D*_ the area, and *D* the effective diameter of the diode. To conclude, theoretically expected responsivity can be calculated by

5 $$ {\mathcal R} [\frac{A}{W}]={R}_{A}\frac{{\partial }^{2}I}{\partial {U}^{2}}{|}_{{U}_{DC}}{\eta }_{opt}{\eta }_{imp}{\eta }_{RC}$$

### N-i-pn-i-p photodiode THz source

We used as a CW THz source an InGa(Al)As based n-i-pn-i-p superlattice potomixer with a broadband logarithmic-periodic antenna^50^. This photomixer was packaged with a fiber-pigtail and a DC-bias connector. THz radiation was generated by photomixing of two tunable telecom lasers which were amplified by a commerical EDFA (erbium-doped fiber amplifier). We used a commercial TeraScan laser system from Toptica Photonics for our experiments. We note here that the resulting THz-bandwidth is exclusively determined by the linewidth of the photomixing lasers.

### Spectroscopy setup

For spectroscopic measurements, a stainless steel gas cell of a 1 m length and polyethylene windows of 5 mm thickness were used. The pressure in the cell can be adjusted between 1 · 10^−3^ mbar and 1000 mbar. For the high resolution spectra a Toptica TeraScan laser system with a linewidth of 1 MHz was used for the photomixing. During the measurements the graphene Schottky diode was operated at zero bias to guarantee a low noise floor. For extracting the transmission of a gas species a reference spectrum was measured with an evacuated cell. The cell was then filled by the invesigated organic compound and the pressure was reduced to the desired level by pumping. The spectrum was measured and subsequently divided by the reference spectrum to receive the transmission of the gas species. These spectra were compared to simulations based on the JPL database.

## Supplementary information

Supplementary Information
