# Supplementary material for: An efficient Terahertz rectifier on the graphene/SiC materials platform
Source: Sci Rep. 2019 Aug 1;9:11205. doi: 10.1038/s41598-019-47606-6 (PMC6671971; doi:10.1038/s41598-019-47606-6)
Supplement: Supplementary file 1 — Supplementary Information [file 41598_2019_47606_MOESM1_ESM.pdf]

Supplementary Information
An efficient Terahertz rectifier on the graphene/SiC materials platform

Maria T. Schlecht[1], Sascha Preu[2], Stefan Malzer[1], and Heiko B. Weber[1,*]

[1]Friedrich-Alexander University of Erlangen-Nürnberg (FAU), Applied Physics, Staudtstr. 7 / A3, 91058 Erlangen, Germany
[2]Department of Electrical Engineering and Information Technology, Technical University Darmstadt, Merckstrasse 25, 64283 Darmstadt, Germany
[*]heiko.weber@fau.de

References

1. Secker, D. *et al.* Resonant vibrations, peak broadening, and noise in single molecule contacts: The nature of the first conductance peak. *Phys. Rev. Lett.* **106**, 3–6, DOI: 10.1103/PhysRevLett.106.136807 (2011).

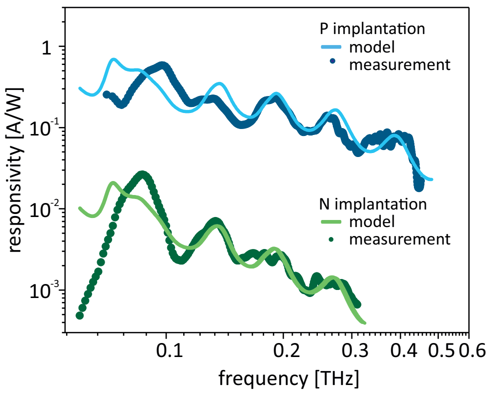

Figure 1. Supplementary Figure S1: THz responsivity of the second phosphorous test device in comparison with the very same nitrogen device that is displayed in figure 2. The responsivity is slightly lower compared to the device described in the main manuscript. The experimental data retrace the calculated characteristics much better. The origin of the deviations in figure 2 in the main manuscript is structural imperfection at one of the antenna arms.

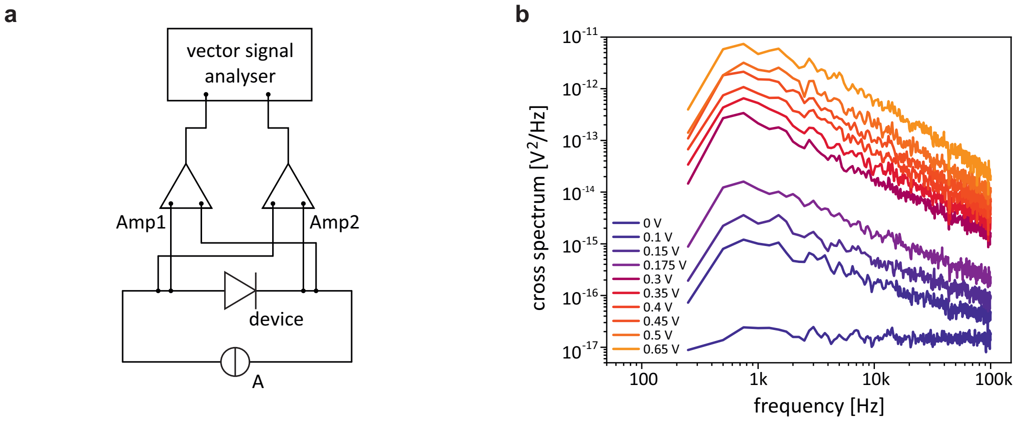

Figure 2. Supplementray Figure S2: Noise cross spectrum of a phosphorous implanted Schottky diode. (**a**) Measurement setup according to[1]. (**b**) The cross spectra (loglog scale) shown are dominated by 1/f noise in the frequency range of the chopping frequency.
